# It's here, monkeypox: A case report

**DOI:** 10.1016/j.jdcr.2022.07.037

**Published:** 2022-08-12

**Authors:** Samavia Khan, Shazli Razi, Babar Rao

**Affiliations:** a Center for Dermatology, Rutgers Robert Wood Johnson Medical School, New Brunswick, New Jersey; b Rao Dermatology, Atlantic Highlands, New Jersey; c Department of Dermatology, Weill Cornell Medicine, New York, New York

**Keywords:** complex medical dermatology, dermatology, infectious disease, monkeypox, pandemic, CDC, Centers for Disease Control and Prevention, PCR, polymerase chain reaction

## Introduction

The monkeypox outbreak has prompted global concern because of poxviridae’s transmissibility through respiratory droplets, intimate contact, and possibly, sexual fluids.^1^ According to the Centers for Disease Control and Prevention (CDC) criteria, monkeypox is suspected when a patient develops a characteristic rash of “deep-seated and well-circumscribed lesions, often with central umbilication; and lesion progression through specific sequential stages: macules, papules, vesicles, pustules, and scabs.”^2^ Alternatively, monkeypox is suspected when there is clinical suspicion and the patient meets 1 of the following epidemiologic criteria within 21 days of symptom onset^2^:

1. Having “close or intimate contact with persons in a social network experiencing monkeypox infections,” such as men who have sex with men
2. Traveling to a country with confirmed cases of monkeypox or “where monkeypox is endemic”
3. Having contact with a person with a similar rash or probable monkeypox
4. Having contact with a dead or live animal that is an African endemic species
5. Having contact with a person with a similar rash or probable monkeypox.

Knowledge of the CDC criteria can promote early detection of monkeypox and a more intentional and safe dermatologic examination. In addition, with this knowledge, dermatologists can become quickly alert to a suspicious exanthem and provide patients with self-isolation instructions to halt further spread. We present our dermatologic approach to a case of suspected monkeypox in New York City and the associated clinical and histopathologic findings.

## Case report

A 31-year-old man developed fatigue and fever after returning from a vacation in Miami, Florida. Two days later, he developed uncomfortable blisters on his entire body, which prompted him to visit an urgent care center, where the lesions were swabbed for polymerase chain reaction (PCR) testing. He was not given specific instructions on what to do until the PCR results were available. Hence, he continued to engage in his daily activities, including attending work and large-scale social events. Because the patient’s lesions did not resolve, he presented at our dermatology practice 4 days later for an evaluation.

**Fig 1:**
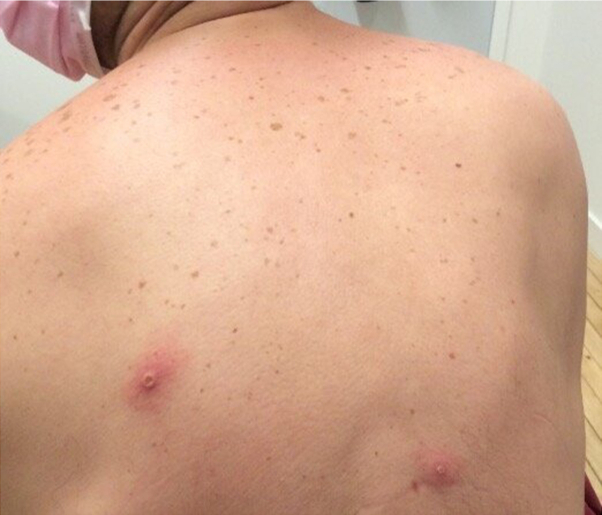
Clinical presentation of monkeypox on the trunk. Pink umbilicated papules measuring 2 to 8 mm in the same stage of development with surrounding erythema.

**Fig 2:**
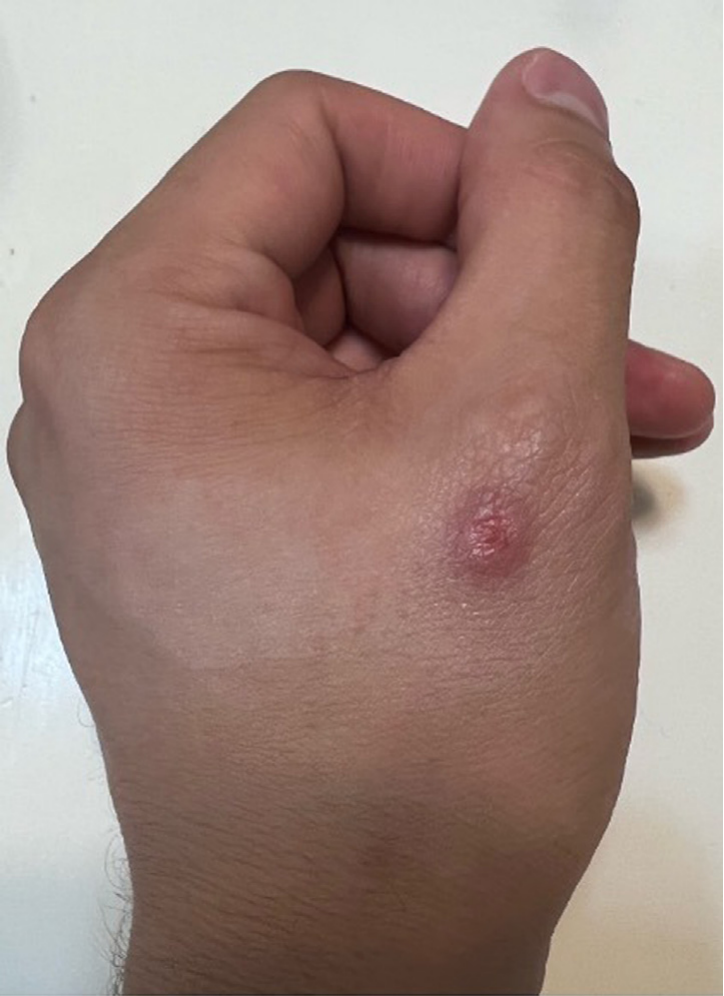
Clinical presentation of monkeypox on the palms and soles. Five to 6 mm wide, pink, deep-seated vesicles with erosion

The patient reported that he was previously “healthy,” with unremarkable medical and surgical history. The patient identified himself as gay, although a detailed sexual history was not obtained. The patient’s vitals were stable, and he was afebrile. Physical examination revealed that the patient had approximately 20 pink umbilicated papules, 2 to 8 mm in diameter, in the same stage of development, with surrounding erythema distributed across the entire body, including the face, trunk, and both arms and legs (Fig 1). He also had approximately 5 to 6 mm wide, pink, deep-seated vesicles with erosion on the palms and soles (Fig 2). The patient had bilateral inguinal lymphadenopathy. No lesions were observed in the mucosal membranes of the mouth or the genitals.

Given his recent travel history and the vesiculopapular exanthem, N95 masks and gloves were worn during the examination. Differential diagnoses included monkeypox, herpes simplex virus, varicella-zoster virus, and syphilis. Herpes simplex virus and varicella-zoster virus often present with smaller-sized lesions that are more anatomically limited in distribution to a single anatomical region or a dermatome. The morphology, distribution, and painful nature of the lesions made primary syphilis less likely. In addition, the acute onset and painful nature of the lesions made lichenoid secondary syphilis less likely. The patient’s recent travel history and clinical presentation met the CDC criteria of “suspected monkeypox,” and he was immediately reported to the CDC Emergency Operations Center and New York City Health Department. The patient was guided to quarantine for at least 3 weeks to prevent further transmission.

**Fig 3:**
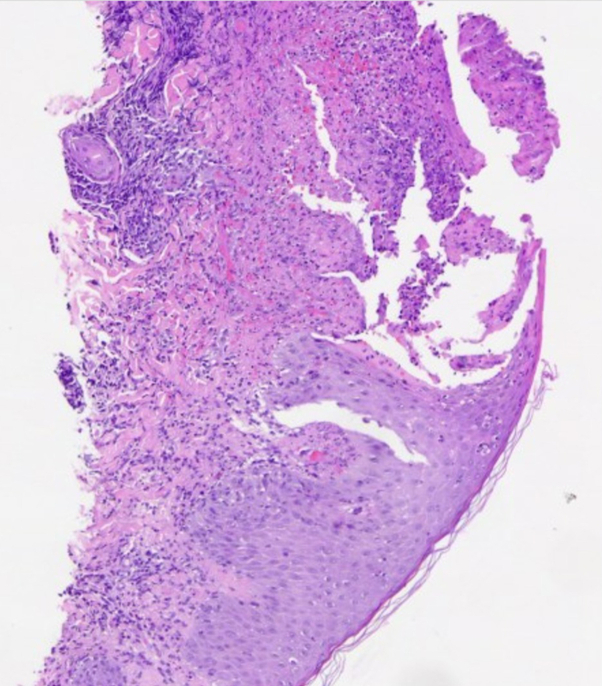
Biopsy of the monkeypox lesion on the trunk. Focal, full-thickness epidermal necrosis and several multinucleated keratinocytes were noted. (Hematoxylin**-**eosin stain; original magnification: ×20.)

**Fig 4:**
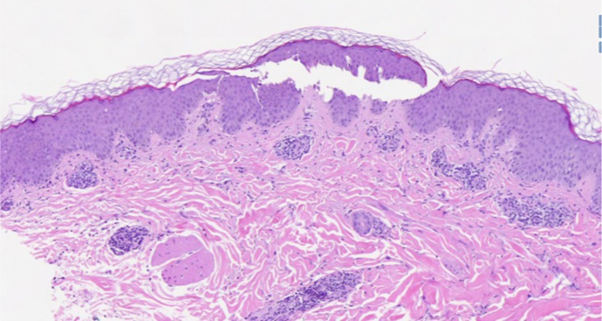
Biopsy of the monkeypox lesion on the trunk. A dense dermal inflammatory infiltrate composed of lymphocytes, neutrophils, and red blood cells extending into the epidermis was noted, consistent with a viral infection. (Hematoxylin**-**eosin stain; original magnification: ×20.)

On histology, focal, full-thickness epidermal necrosis and several multinucleated keratinocytes were noted (Fig 3). A dense dermal inflammatory infiltrate composed of lymphocytes, neutrophils, and red blood cells extending into the epidermis was noted, consistent with viral infection (Fig 4). Two swabs from 2 lesions were collected using dry polyester swabs with a wood shaft and sent for real-time PCR, with no transport media. Monkeypox DNA was confirmed via real-time PCR testing. Within the next 2 weeks, the patient’s symptoms of fatigue and skin lesions self-resolved, with no evidence of postinflammatory hyperpigmentation.

## Discussion

High clinical suspicion for monkeypox is warranted in patients who present with an acute onset vesiculopustular/vesiculopapular rash with recent travel history or men who have sexual contact with other men.^2^ Dermatologists approaching a suspected case of monkeypox infection should use a gown, gloves, eye protection (goggles or face shield), and a National Institute for Occupational Safety and Health–approved particulate respirator equipped with N95 filters or higher.^2^ Patients should be restricted to a single-person room and should also wear personal protection equipment. Specimens should be labeled clearly in sterile, plastic, leak-proof containers and shipped for analysis promptly. Dermatologists should follow their institutional protocols and should immediately consult the CDC Emergency Operations Center or their local health departments if the monkeypox virus is suspected.

While the global impact of the COVID-19 pandemic continues to linger, the emergence of the monkeypox outbreak poses an unsettling public health threat. The World Health Organization declared monkeypox a global health emergency on July 23, 2022, because of a staggering uptick of cases, with a recent case fatality rate of 3% to 6%.^3^ As of July 25, 2022, there were 3846 confirmed cases of monkeypox in the United States, with more than a quarter of the cases being from the state of New York alone.^2^ Because of the outbreak’s epidemiologic burden on the community of men who have sex with men, appropriate public health awareness and mitigation strategies must be urgently developed, which will advance the health and well-being of this community without perpetuating stigma. By strategically approaching suspected monkeypox exanthems with the goals of containment and patient/team safety, dermatologists can play an invaluable role in preventing further spread of this zoonotic disease.

## Conflicts of interest

None disclosed.
